# Editorial Correspondence

**Published:** 1871-09

**Authors:** John C. Drake

**Affiliations:** Thomaston, Ga.


The object we desire to subserve by the Editorial Department of THE COMPANION is, that the principles, interests and dignity of the Profession may be discussed, for the union of all true physicians, and for the up-building of a common calling. We earnestly desire to have the views of the Profession upon every point touching the well-being of the Profession—upon ethics, fee-bills, medical education, colleges, etc., etc., that our calling may be exalted, and the best interests, both of the Profession and the people, may be protected and promoted. Whenever, therefore, we receive such letters as those below, we will most cheerfully surrender our space for their publication.

Dr. John C. Drake, one of the Associate Editors of THE COMPANION, writes:

THOMASTON, GA., AUGUST 1, 1871.

DRS. POWELL & GOLDSMITH:

*My Dear Sirs*—I have been gratified to see that you had started a journal of the right stamp, and one which I hope will take high grounds and become the organ of the Profession in the State. This, at the present juncture, has become a necessity, and unless something is done to stop the *innovations* and *quackery*, and uphold, to the utmost, the Medical Ethics, honor and high tone of the Profession, it will sink so low, that the masses even will cease to do it reverence. The very high position which the Profession once occupied, I regret to say, is fast giving way, and unless something is done to sustain it, will become almost a reproach. I can well remember the time when it was an honor to be a physician, but now anybody can be one. I attribute much of this to the course which has been pursued by the various colleges, (in effort at numbers) in turning out upon the country, men unfit and unworthy of the name. Such a course has had the effect of driving from the Profession the best talent of the country, who were unwilling to wire work and grapple with quacks, and the irregularities of unworthy so-called doctors.

I have looked on this state of things with deep regret and mortification, and the hope I once had, that the State and National Medical Associations, would take high grounds for the salvation of the Profession, I am sorry to say, is fast giving away, for the same state of things exist everywhere. Unworthy and designing men have taken control. This should not be so. How can this be altered? To this end I go for taking high grounds in Ethics, and let no man pass our colleges and societies with the M. D. attached to his name, unless he possesses every qualification—morality, sobriety, integrity, high sense of honor, and thorough education, medical and literary. Let these be the requisites, the Shibboleth of every applicant for the degree or for membership of medical societies.

I am much pleased with the appearances of THE GEORGIA MEDICAL COMPANION, and hope it may become the organ of the Profession in the State, and I can see no good reason why it should not be. I think the Profession should take the matter in hand and give every assistance needful.

I am very truly yours, JOHN C. DRAKE.

